# Varicella Zoster Aseptic Meningitis Presenting as an Atypical Mucocutaneous Eruption Involving All Three Divisions of the Trigeminal Nerve

**DOI:** 10.7759/cureus.20988

**Published:** 2022-01-06

**Authors:** Maja Magazin, Nicholas B Castner, Gina Askar, Budder Siddiqui

**Affiliations:** 1 Ophthalmology, Augusta University Medical College of Georgia, Augusta, USA; 2 Department of Dermatology, Stony Brook University, Stony Brook, USA; 3 Infectious Disease, Augusta University Medical College of Georgia, Augusta, USA

**Keywords:** multi-dermatomal, aseptic meningitis, trigeminal nerve, herpes zoster, varicella zoster

## Abstract

Herpes zoster involving all three divisions of the trigeminal nerve is extremely rare and may pose a diagnostic challenge, especially in young and immunocompetent patients. We present a unique case of herpes zoster involving all three divisions of the trigeminal nerve and illustrate that this uncommon eruption can be a presenting sign of varicella zoster aseptic meningitis. This case emphasizes the importance of fundamental morphology recognition, particularly its ability to aid in clinical diagnosis and its potential to decrease patient morbidity and mortality by expediting the initiation of appropriate treatment.

## Introduction

Herpes zoster is a dermatomal, painful, vesicular rash caused by the reactivation of the varicella zoster virus (VZV) [1]. It is most frequently seen in the elderly or immunocompromised [2]. Other risk factors for reactivation include physical trauma, dental manipulation, high stress, and radiation. The trigeminal nerve is involved in about 15% of cases with the ophthalmic branch affected about 20 times more often than the maxillary or mandibular branch [1-3]. Multi-dermatomal involvement of the trigeminal nerve is extremely rare with a recent review identifying only six published cases [2].

Herein, we describe the first known presentation of multi-dermatomal herpes zoster involving all three branches of the trigeminal nerve with unilateral oral vesicles and aseptic viral meningitis in a young, immunocompetent adult. This case highlights the importance of analyzing the morphology and distribution of a dermatologic process to not miss an atypical aggressive case of VZV in an otherwise healthy individual and prevent the possible morbidity and mortality associated with delayed diagnosis.

## Case presentation

**Figure 1:**
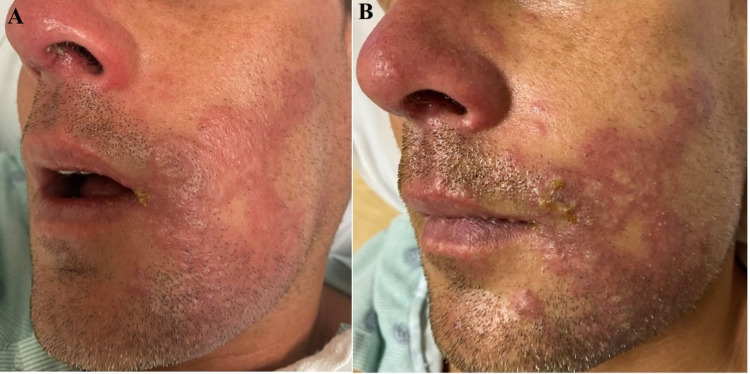
Herpes Zoster of the Trigeminal Nerve. A: Patient on day one of admission with multiple erythematous, edematous, well-demarcated plaques to the left jawline, cheek, and nose. B: On day two of admission, the plaques developed vesicles and pustules.

A 33-year-old previously healthy male presented with oral pain and a painful left-sided facial rash of two days duration. The patient reported a left-sided headache with subjective fevers, body aches, night sweats, and odynophagia. He had an uncomplicated left upper molar extraction one month prior. Upon presentation, skin examination revealed erythematous, edematous, well-demarcated plaques to the left jawline, cheek, eyebrow, and nose (Figure 1).

**Figure 2:**
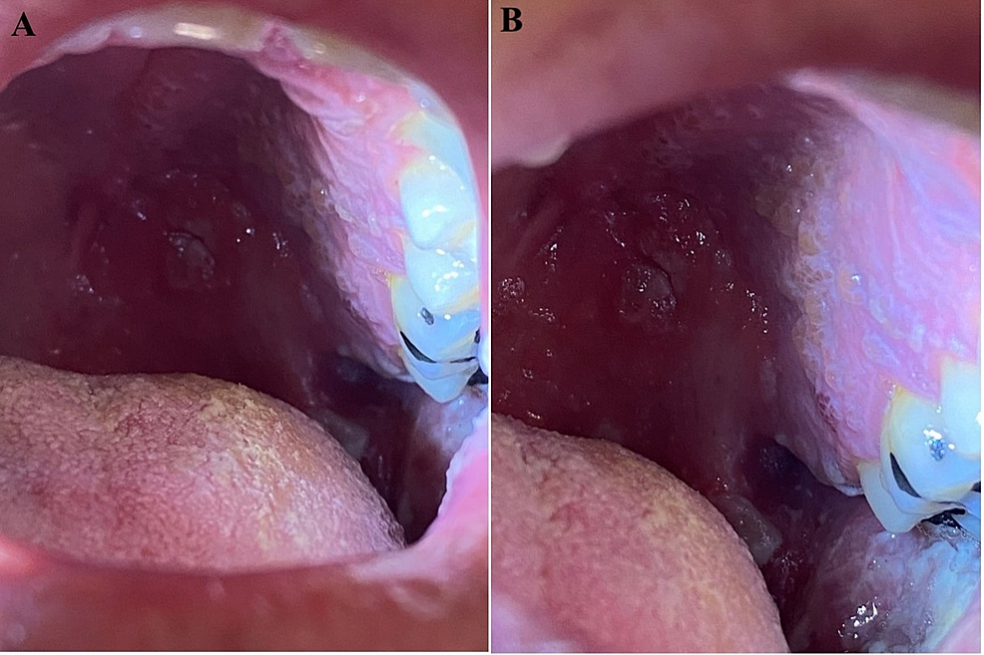
Intraoral Vesicular Eruption in Herpes Zoster. A,B: Intraoral examination on the day of admission demonstrating multiple coalescing vesicles to the left buccal mucosa and soft and hard palate, indicating the involvement of the V2 and V3 branch of the trigeminal nerve. The vesicular eruptions strictly obeyed the midline and raised suspicion for herpes zoster.

Oral examination showed multiple coalescing vesicles to the left buccal mucosa and soft and hard palate, all strictly obeying the midline (Figure 2). A lumbar puncture was performed due to concerns for central nervous system involvement, and cerebrospinal fluid (CSF) analysis suggested a viral etiology with a lymphocytic pleocytosis and elevated protein. Polymerase chain reaction (PCR) of the facial plaques revealed VZV DNA. PCR from the spinal fluid analysis was also positive for VZV DNA, establishing a diagnosis of multi-dermatomal trigeminal nerve herpes zoster with associated aseptic viral meningitis. In addition, the otoscopic and ophthalmological examination was unremarkable, cranial nerves were intact, and there was no evidence of neurological deficits. Human immunodeficiency virus testing was negative. The patient was treated with a two-day course of high-dose intravenous acyclovir with a transition to a 10-day course of oral valacyclovir. The patient subsequently developed symptoms suggestive of postherpetic neuralgia for which amitriptyline and gabapentin were initiated with resulting improvement.

## Discussion

Given its rarity, a multi-dermatomal vesicular rash may pose a diagnostic challenge, especially in young and immunocompetent patients. The patient in this case report was found to have multi-dermatomal herpes zoster of the trigeminal nerve with disseminated disease to the central nervous system, which has not previously been reported in an immunocompetent individual. However, aseptic meningitis with an associated abdominal herpes zoster eruption has been observed, highlighting the ability of herpes zoster to present with concomitant central nervous system invasion [4]. The initial differential diagnosis in the current case was broad. However, the sharp demarcation of the unilateral oral vesicles raised the clinical suspicion for herpes zoster and allowed for early diagnosis of an atypical presentation. As seen in this case, the presence of vesicular lesions on the soft and hard palate can indicate the involvement of the maxillary branch of the trigeminal branch, while the involvement of the mandibular branch can manifest as vesicular lesions to the buccal mucosa [2]. Careful examinations of the oral mucosa can be crucial in establishing an early diagnosis and initiating aggressive treatment.

There have been only a few published cases of herpes zoster affecting all three branches of the trigeminal nerve, although none were associated with disseminated central nervous system infections [1,5,6]. Similar to our case, Naveen et al. presented a young, immunocompetent male with herpes zoster of all three divisions of the trigeminal nerve with the involvement of the hard palate, further highlighting the importance of examining the oral mucosa [6]. Although the patient in the current case did not have underlying immunosuppression, he did undergo an uncomplicated dental extraction of the left upper molar one month prior. Herpes zoster following dental manipulation is a rare complication, with previous case studies reporting vesicular eruptions less than a week after the procedure [7,8]. While it is possible that dental trauma may have triggered multi-dermatomal reactivation in the current case, the delayed timing and associated aseptic meningitis in an immunocompetent individual presents a unique consideration.

## Conclusions

This case reemphasizes the importance of analyzing distribution, morphology, and arrangement in addition to conducting a thorough examination, including mucous membranes, when evaluating a patient with a new eruption. Clinicians should maintain high clinical suspicion of central nervous system dissemination in all patients with systemic symptoms, especially in the setting of multi-dermatomal eruptions, as such aggressive presentations will require prompt treatment with intravenous acyclovir. Viral PCR from either cutaneous lesions or CSF can take several days to result. Clinical diagnosis made on the basis of morphology, even in rare or atypical cases, has the ability to decrease the morbidity associated with delayed diagnosis of VZV.
